# Comparison of melatonin with growth factors in promoting precursor cells proliferation in adult mouse subventricular zone

**DOI:** 10.17179/excli2016-606

**Published:** 2016-12-21

**Authors:** Areechun Sotthibundhu, Kasima Ekthuwapranee, Piyarat Govitrapong

**Affiliations:** 1 Center for Neuroscience, Faculty of Science, Mahidol University, Bangkok, Thailand; 2 Chulabhorn International College of Medicine, Thammasat University, Patumthani, 12120, Thailand; 3 Physical therapy, Srinakharinwirot University, Ongkharak, Nakhonnayok 26120, Thailand; 4 Research Center for Neuroscience, Institute of Molecular Biosciences, Mahidol University, Salaya, Nakornpathom, Thailand; 5 Chulabhorn Graduate Institute, Kamphaeng Phet 6 Road, Lak Si, Bangkok 10210, Thailand

**Keywords:** epidermal growth factor, extracellular signal-regulated protein kinase, melatonin, mitogen-activated protein kinase, neural stem cells, subventricular zone

## Abstract

Melatonin, secreted mainly by the pineal gland, plays roles in various physiological functions including protecting cell death. We showed in previous study that the proliferation and differentiation of precursor cells from the adult mouse subventricular zone (SVZ) can be modulated by melatonin via the MT1 melatonin receptor. Since melatonin and epidermal growth factor receptor (EGFR) share some signaling pathway components, we investigated whether melatonin can promote the proliferation of precursor cells from the adult mouse SVZ via the extracellular signal-regulated protein kinase /mitogen-activated protein kinase (ERK/MAPK) pathways in comparison with epidermal growth factor (EGF). Melatonin-induced ERK/MAPK pathways compared with EGF were measured by using *in vitro* and *vivo* models. We used neurosphere proliferation assay, immunocytochemistry, and immuno-blotting to analyze significant differences between melatonin and growth factor treatment. We also used specific antagonist and inhibitors to confirm the exactly signaling pathway including luzindole and U0126. We found that significant increase in proliferation was observed when two growth factors (EGF+bFGF) and melatonin were used simultaneously compared with EGF + bFGF or compared with melatonin alone. In addition, the present result suggested the synergistic effect occurred of melatonin and growth factors on the activating the ERK/MAPK pathway. This study exhibited that melatonin could act as a trophic factor, increasing proliferation in precursor cells mediated through the melatonin receptor coupled to ERK/MAPK signaling pathways. Understanding the mechanism by which melatonin regulates precursor cells may conduct to the development of novel strategies for neurodegenerative disease therapy.

## Abbreviations

BDNF: brain-derived neurotrophic factor; bFGF: basic fibroblast growth factor; EGF: epidermal growth factor; EGFR: epidermal growth factor receptor; ERK: extracellular signal-regulated kinase; MT: melatonin receptor; PKA: protein kinase A; PKC: protein kinase C; PLC: phospholipase C; MAP: mitogen-activated protein; MAPK: mitogen-activated protein kinase; NAS: N-acetyl serotonin; SGZ: subgranular zone; SVZ: subventricular zone

## Introduction

Two active areas of adult brain which still have cell proliferation and differentiation are the subgranular zone (SGZ) in the dentate gyrus of the hippocampus and the anterior part of the subventricular zone (SVZ) lining the lateral ventricle (Altman and Das, 1965[2]; Altman, 1969[1]). The proliferation and differentiation of adult precursor cells are modulated by several molecules, such as growth factors and hormones, under physiological and pathological conditions (Lledo et al., 2006[31]). Precursor cells obtained from the adult SVZ and cultured in epidermal growth factor (EGF)-containing medium grow to form neurospheres that are self-renewing and multipotent (Ayuso-Sacido et al., 2010[3]; Doetsch et al., 2002[15]; Shi et al., 2008[49]). EGF was discovered by Cohen and Elliott (1989[10]). Subsequent studies demonstrated that EGF is a usual mitogenic factor that stimulates the generation of different types of cells such as fibroblasts and epithelial cells (Cohen and Elliott, 1963[11]). Progenitor cells in both the embryonic and postnatal SVZ divide in response to EGF ligand and EGF receptor (EGFR) expression, and they retain the ability to differentiate into neurons and glial cells (Gritti et al., 1999[17]; Reynolds et al., 1992[42]; Weickert et al., 2000[56]). EGFR signaling influences vary cellular mechanisms involved in neurogenesis, including neural cell survival, proliferation, differentiation and migration, by activating the mitogen-activated protein (MAP) kinase (MAPK) signaling cascade via the extracellular signal-regulated kinase (ERK1/2) (Carreira et al., 2010[8]; Shi et al., 2008[49]). 

Melatonin (5-methoxy-N-acetyltryptamine) secreted mainly by the pineal gland, controls several physiological functions (Stehle et al., 2011[52]). Melatonin as well as its metabolites plays anti-apoptotic free radical-scavenging properties (Hardeland et al., 2012[18]). The intermediate precursor of melatonin, N-acetyl serotonin (NAS), enhanced proliferation and differentiation in hippocampal cells through brain-derived neurotrophic factor (BDNF) receptor TrkB (Jang et al., 2010[20]; Sompol et al., 2011[50]; Tosini et al., 2012[54]; Yoo et al., 2011[59]). Extending demonstration also suggests that melatonin might be an attractive agent in the neurogenesis context. Melatonin enhanced neurogenesis in embryos (Kong et al., 2008[27]; Moriya et al., 2007[36]). Our previous study showed that melatonin facilitated proliferation in precursor cells from the adult mouse SVZ and the adult rat SGZ via the melatonin receptor (MT1) (Sotthibundhu et al., 2010[51]; Tocharus et al., 2014[53]). Consistent with this view, neuritogenesis and differentiation in neuroblastoma cells can be influenced by melatonin *in vitro* through a mechanism that involves the activation of ERK via the MT1 receptor (Witt-Enderby et al., 2000[58]). Melatonin promoted human mesenchymal stem cell differentiation into osteoblasts through the formation of MT2/MEK/ERK1/2 complexes, causing osteogenic gene expression (Radio et al., 2006[40]; Sethi et al., 2010[48]). 

Since melatonin and EGF share some similar effects, we compared the proliferative effect and the signaling transductions of melatonin and EGF in the adult mouse SVZ precursor cells by using *in vitro* and *in* *vivo* model studies. 

## Materials and Methods

### Drug administration

Sixteen male 8-week-old C57BL6 mice were purchased from the National Experimental Animals Center of Mahidol University, Salaya Campus, Thailand. The experiment procedures conformed under ethical guideline for animal research which is approved by the Mahidol University Animal Care and Use Committee (MU-ACUC). Drug administration was performed according to our previously described protocol (Mukda et al., 2009[37]). Briefly, at eight weeks, C57BL6 mice were randomly appointed to either saline or melatonin groups. Each group comprised of 8 mice. The animals were injected subcutaneously with saline or melatonin (5 mg/kg body weight; M5250, Sigma-Aldrich, St. Louis, MO, USA) once daily (at 10:00 a.m. to obviate endogenous outcome of melatonin) for 7 consecutive days. In our previous study, 5 mg/kg body weight melatonin was the minimal effective dose (Mukda et al., 2009[37]). Melatonin is very low at 10 a.m., therefore this time point is suitable to detect any change. The C57BL6 mouse is a proper stain to study the role of exogenous melatonin on the progenitor cell proliferation. This stain expresses both MT1 and MT2 melatonin receptors, although it synthesizes low level of melatonin compared with other stains of mice (Dubocovich et al., 2005[16]; Roseboom et al., 1998[44]). In this case, the existing melatonin receptor may become supersensitive thus this makes this animal species suitable for studying its activity. Animals were sacrificed 24 hrs after the last treatment. Brains were immediately removed and dissected. The SVZ was dissected for Western blot analysis.

### Neurosphere proliferation assay

Adult neural precursors were obtained from the adult mouse SVZ and cultured as floating aggregates (neurospheres), as previously described (Sotthibundhu et al., 2010[51]). Briefly, SVZ tissue was dissociated in 0.05 % trypsin (25300-054, Invitrogen, San Diego, CA, USA), plated in 24-well plates at a density of 1800 cells per well or plated in T25 flasks at a density of 0.5x10^4^ cells/ml. The cells were cultured at 37 °C in 5 % CO_2_ for 7 days in serum-free neurosphere medium containing NeuroCult (05701, Stem Cell Technologies, Vancouver, Canada) with or without 10 ng/ml bFGF (11123149001, Roche, Mannheim, Germany) and 20 ng/ml EGF (356052, BD Biosciences, Franklin, NJ, USA). Melatonin (100 µM melatonin dissolved in 40 % ethanol for 1 µM melatonin as a final concentration) was added to SVZ cultures at day 0. In some experiments, SVZ cultures were also treated with either luzindole (L2407, Sigma-Aldrich; 100 µM luzindole dissolved in DMSO for a final concentration as 1 µM luzindole) or U0126 (U120, Sigma-Aldrich; 23.5 µM U0126 dissolved in DMSO for a final concentration of 1 µM U0126). At the end of the 7-day proliferation period, the size and number of neurospheres per well was defined.

### Immunocytochemistry and quantitative immunofluorescence determination 

The effect melatonin on the expression of nestin was investigated by transferring neurospheres to cover slip coated with poly-L-ornithine (P4957, Sigma-Aldrich) and laminin (CC095, Millipore, Bedford, MA, USA). After 1 day of incubation in 37 °C, neurospheres were fixed with 4 % paraformaldehyde for 20 min at room temperature and washed with PBS 3 times before permeabilized using 1 % Triton X-100 for 10 min. Non-specific binding was blocked with 5 % normal goat serum in PBS containing 1 % Trion X for 10 min before incubating with the mouse monoclonal antibody against nestin (1:200, MB353, Millipore) and rabbit polyclonal antibody against Ki67 (1:200, AB9260, Millipore) overnight at 4 °C. The coverslips were washed three times with PBS and incubated with Alexa Fluor® 488 Goat Anti-Rabbit IgG (1:2000, ab150077, Abcam, Cambridge, MA, USA), Alexa Fluor® 568 Goat Anti-Mouse IgG fluorescent dye (1:2000, ab175473, Abcam), and DAPI nuclear stain from Invitrogen (1:2000, 62248, Invitrogen) for 2 hrs at room temperature. The coverslips were mounted with antifade reagent (H1000, Vectashield, Vector Laboratories, Burlingame, CA, USA) after washing with PBS. The immunoreactivity was visualized using a confocal laser scanning microscope (Olympus Fluoview FV10i, Tokyo, Japan).

To quantify the intensity of Ki67 and DAPI staining, images were captured at 10x or 60x magnification from at least five randomly selected fields for each of four independent experiments. ImageJ software (NIH) was used to compute the fluorescence intensity.

### Western blot analysis

Following experimental treatments, the cell pellet and SVZ tissue were resuspended in lysis buffer with 1 % protease inhibitor and 1 % phosphatase inhibitor and sonicated for 10 s twice. Lysate was then centrifuged at 12,000 × *g* for 15 min at 4 °C, and the supernatant was used for western blot processing. After the protein concentration was determined by the method described by Lowry et al. (1951[32]), the samples were denatured in sample buffer at 95 °C for 5 min. Protein was loaded onto a 10-12 % SDS-PAGE gel and then electrophoretically transferred to a polyvinylidenedifluoride (PVDF) membrane (Amersham Biosciences, Piscataway, NJ, USA). The transfer efficiency was checked by Ponceau-S red staining (Bio-Rad Laboratories, Hercules, CA, USA). The membranes were washed with Tris-buffered saline containing 0.1 % Tween-20 (TBST) for 5 min, incubated in blocking buffer for 1 hr at room temperature and then incubated overnight at 4°C with rabbit polyclonal antibody against c-Raf (1:1,000, 9422, Cell Signaling, Danvers, MA, USA), rabbit polyclonal antibody against p-c-Raf (1:1,000, 9427, Cell Signaling), mouse monoclonal antibody against ERK1/2 (1:5,000, SC514302, Santa Cruz, CA, USA), mouse monoclonal antibody against p-ERK1/2 (1:5,000, SC81492, Santa Cruz), rabbit polyclonal antibody against c-Myc (1:1,000, SC40, Santa Cruz) or rabbit polyclonal antibody against p-c-Myc (1:1,000, SC8000-R, Santa Cruz). The membranes were washed three times for 5 min each with TBST, incubated in HRP-conjugated anti-mouse IgG antibody (1:10,000, 7076, Cell Signaling) or anti-rabbit IgG antibody (1:10,000, 7074, Cell Signaling) for 1 hr at room temperature and washed in TBST. Finally, the membranes were visualized by enhanced chemiluminescence using ECL^TM^ Prime (RPN2232, Amersham Biosciences) and the membrane was exposed on X-ray film (Kodak, Rochester, NY, USA). The immunoblot band densities were normalized with its unphosphorylated protein and quantified using a densitometer in the Scion image analysis program (National Institutes of Health, Bethesda, MD, USA).

### Data analysis and statistical methods

The data from all experiments were expressed as the mean ± S.E.M. and compared using a one-way analysis of variance (ANOVA) followed by the Tukey-Kramer test or the independent samples *t*-test using GraphPad Prism version 5. Significance was assumed when *P*-values were less than 0.05. 

## Results

### Effect of melatonin on nestin and Ki-67 immunoreactivity

**Figure 1:**
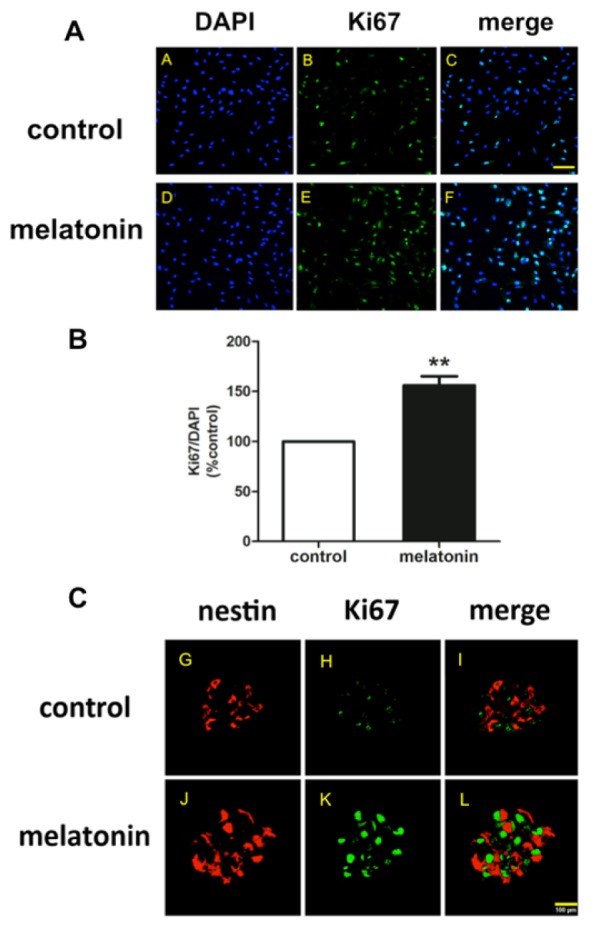
The neurosphere staining with anti-Ki67 (green) and anti-nestin (red) antibodies. Progenitor cells derived from adult SVZ were cultured in the medium containing growth factor in the absence and presence of 1 µM melatonin. Neurospheres were stained with anti-Ki67 and DAPI (A), and with anti-nestin antibodies (C). (B) For quantitative immunofluorescence analysis of Ki-67/DAPI-positive cells, images were captured from at least five randomly selected fields and were analyzed using ImageJ software. T-test was performed for statistical analysis (** denote a significant difference at P < 0.01 compared to the control group. The results were expressed as mean ± S.E.M of four independent experiments and scale bar = 50 (A-F) and 100 µm (G-L).

In order to demonstrate that melatonin is able to increase the proliferation of the progenitor cells derived from adult subventricular zone, the neurospheres were stained using immunocytochemistry method with Ki-67, a proliferating maker (green) and nestin, a multipotential neural stem cell marker (red). The results (Figure 1A, C(Fig. 1)) showed that neurospheres in the proliferative condition revealed positive signal of proliferating marker, Ki-67. The results also indicated that in the cluster of same neurospheres co-localized with nestin protein as shown by red color around the spheres. Ki-67 antigen is expressed during active phase of the cell cycle, whereas the resting phase lacks Ki-67 expression (Kee et al., 2002[24]). While bromodeoxyuridine (BrdU) is expressed during only DNA replication, which could be happened in DNA damage and repair processes, Ki-67 is considered as a suitable marker for study cells in proliferation state. In this study, Ki67 expression was used to detect alterations in proliferation. As shown in Figure 1B(Fig. 1), melatonin increased the ratio of Ki67/DAPI to 155.0 ± 4.4 % of the control treatment (P < 0.01). The result revealed that melatonin treatment increased the expression of Ki-67 and nestin when compared to control. This result indicated that 1 µM melatonin increased cell proliferation as well as the immature neurons. 

### Comparing melatonin with other trophic factors (EGF, bFGF) in promoting precursor cell proliferation 

**Figure 2:**
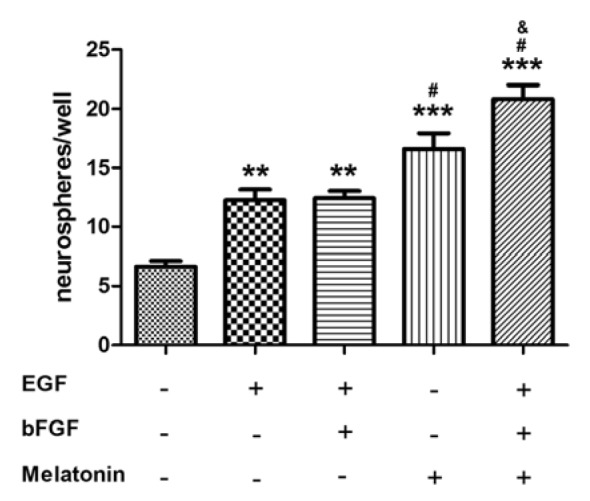
The proliferative effect of growth factors and melatonin on the adult mouse SVZ precursor cells. The numbers of neurospheres per well generated from freshly dissociated SVZ cells plated in the medium in the presence or absence of 20 ng/ml EGF, 10 ng/ml bFGF, and 1 µM melatonin are shown. The neurospheres were formed after culture for 7 days. ANOVA was performed for statistical analysis (** and *** denote statistical significance at *P *< 0.01 and < 0.001, respectively, compared with the control without growth factors, # denotes statistical significance at P < 0.05 compared between melatonin alone versus the combination of two growth factors, and statistical significance at P < 0.05 compared between the combination of two growth factors plus melatonin versus the combination of two growth factors, & denotes statistical significance at P < 0.05 compared between melatonin alone versus the combination of two growth factors plus melatonin). The results are expressed as mean ± S.E.M. of four independent experiments.

To test whether melatonin can act as trophic factor similar to EGF, neurosphere cultures were established with cells isolated from adult mice SVZ area and cultured in the presence or absence of the growth factors. After 7 days of precursor proliferation period, the total number of neurospheres in each culture was counted. The number of neurospheres generated in medium without growth factor (control) was 6.7 ± 0.5 numbers/well (Figure 2(Fig. 2)). The number of neurospheres generated in cultured condition carrying 20 ng/ml EGF, EGF plus 10 ng/ml bFGF, 1 µM melatonin, or melatonin plus EGF and bFGF significantly increased, compared with the control without growth factors (Figure 2(Fig. 2)). No difference in numbers of spheres was found between EGF versus EGF plus bFGF groups. This result was supported from Moriya et al. (2007[36]) which showed the proliferation of neural stem cells completely depends on EGF. Melatonin alone significantly increased the formation of neurosphere when compared with EGF plus bFGF. A significant increase in proliferation was observed when two growth factors and melatonin were used simultaneously compared with EGF plus bFGF (p < 0.01) or melatonin alone (p < 0.01). This suggested the synergistic effect occurred.

### Melatonin increases precursor cell proliferation via the ERK/MAPK pathway

**Figure 3:**
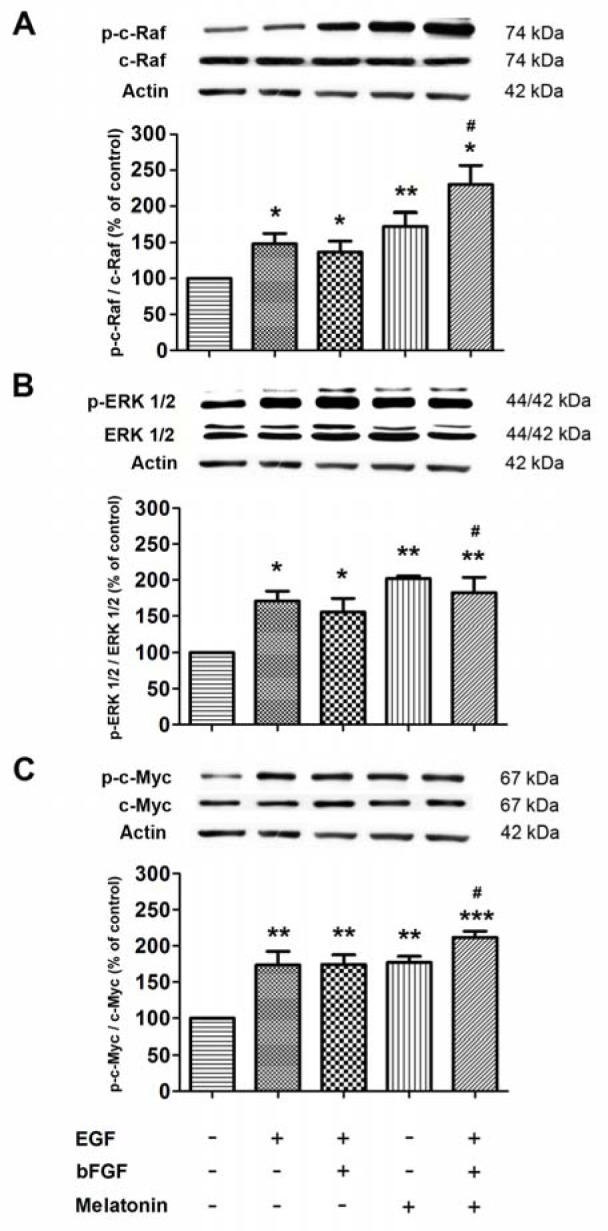
The effect of growth factors and melatonin on signaling protein expressions. Neurospheres were derived from the adult mouse SVZ and cultured in the presence or absence of 20 ng/ml EGF, 10 ng/ml bFGF, and 1 µM melatonin. The levels of p-c-Raf, pERK1/2 and p-c-Myc were normalized with c-Raf, ERK1/2 and c-Myc, respectively, and quantified using densitometry. ANOVA was performed for statistical analysis and values are expressed as mean ± S.E.M. of four independent experiments (*, ** and *** denote statistical significance at *P *< 0.05, < 0.01 and < 0.001, respectively, compared with the control, # denotes statistical significance at P < 0.05 compared between two growth factors versus the combination of melatonin plus two growth factors).

Because the main proliferative pathway of precursor cells is triggered by the activation of EGFR through the ERK/MAPK signaling cascade with the subsequent activation involved with cell cycle modulation both transcription factors and modification of proteins, we hypothesized that melatonin treatment may act via the ERK/MAPK pathway. Therefore, we investigated the expression of signaling molecules in neurospheres derived from the adult mouse SVZ and treated with growth factors and/or melatonin using Western blot analysis. Experiments in which EGF and melatonin were added separately *in vitro *(Figure 3(Fig. 3)). The result showed that melatonin significantly increased the phosphorylation of c-Raf, ERK1/2 and c-Myc and EGF significantly increased phosphorylation of c-Raf, ERK1/2 and c-Myc when compared with control. Two growth factors plus melatonin significantly increased c-Raf, ERK1/2 and c-Myc when compared with EGF + bFGF. The effects of the combination of melatonin plus two growth factors were higher than those effects from growth factor combination. The present result suggested the synergistic effect of melatonin and growth factors on activating the ERK/MAPK pathway. 

**Figure 4:**
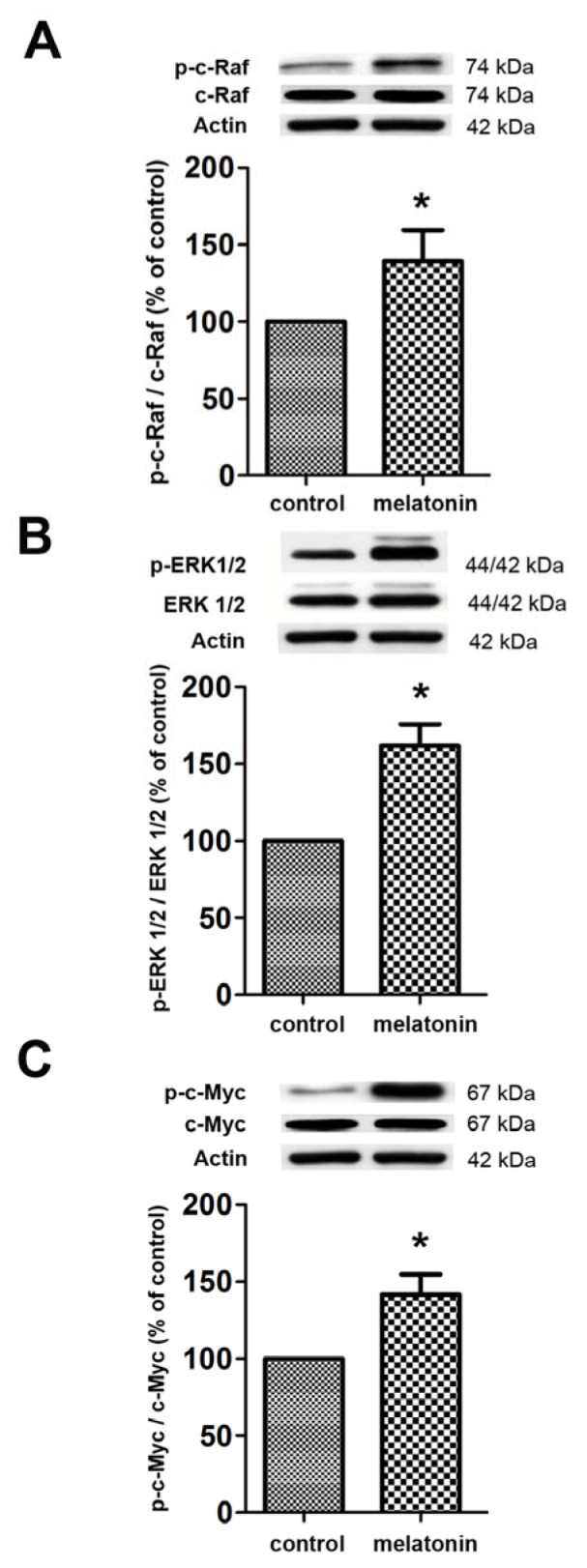
The effect of melatonin on signaling protein expressions *in vivo*. Animals were treated with saline or melatonin for 7 consecutive days. The levels of p-c-Raf, pERK1/2 and p-c-Myc were normalized with c-Raf, ERK1/2 and c-Myc, respectively, and quantified using densitometry. ANOVA was performed for statistical analysis and values are expressed as mean ± S.E.M. from 4 mice per group (* denotes statistical significance at *P *< 0.05, compared with the control).

In addition, we carried out the *in vivo* experiment, melatonin was injected subcutaneously (Figure 4(Fig. 4)). The *in vivo* study also showed that melatonin was able to increase the p-cRaf, p-ERK1/2 and p-c-Myc when compared with the control group. 

### The effect of luzindole, an antagonist of melatonin receptor and U0126, a MEK1/2 inhibitor, on the proliferative effect of melatonin 

**Figure 5:**
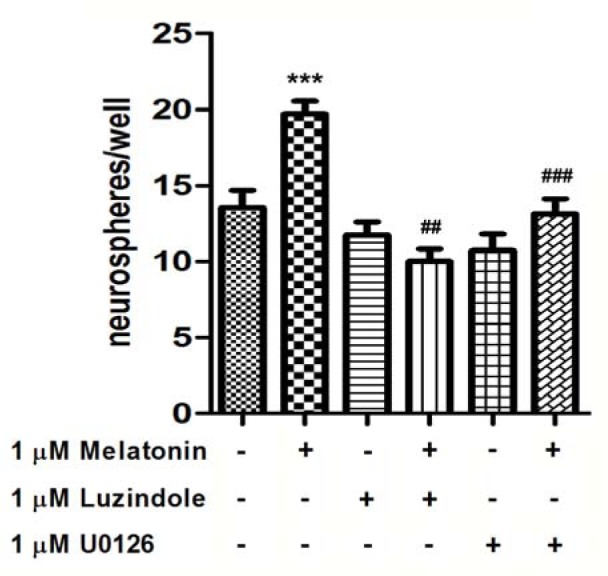
The effect of luzindole, an antagonist of melatonin receptor and U0126, a MEK1/2 selective inhibitor, on the melatonin-induced increase in the proliferation of precursor cells from the SVZ. Neurospheres were generated from freshly dissociated SVZ cells plated in medium containing growth factors in the presence of 1 µM melatonin, 1 µM luzindole and/or 1 µM U0126, as indicated. ANOVA was performed for statistical analysis and the results are expressed as mean ± S.E.M. of four independent experiments (* denotes statistical significance at *P *< 0.001 compared with the control; ## and ### denote statistical significance at *P *< 0.01 and < 0.001, respectively, compared with melatonin-treated cells).

ERK/MAPK signaling pathways are up-regulated upon the activation of various types of receptors (Balmanno and Cook, 2009[5]; Roudabush et al., 2000[46]; Wells, 1999[57]). We examined the involvement of melatonin receptors in the melatonin-induced activation of the ERK/MAPK signaling pathway whether this signaling pathway underlies the proliferative consequence of melatonin on precursor cells from the adult mouse. The total of neurospheres isolated from the SVZ incubated in medium without melatonin (control) was 13.6 ± 1.1 numbers/well. One micromolar melatonin significantly extended the total of neurospheres. When applied alone, 1 µM luzindole or 1 µM U0126 had no significant effect on cell proliferation. Pre-incubation with 1 µM luzindole prior to melatonin, the proliferative effect of melatonin was abolished, and pre-incubation with 1 µM U0126 prior to melatonin significantly abolished the melatonin effects (Figure 5(Fig. 5)). The results indicated that melatonin promotes cell proliferation via melatonin receptor through ERK/MAPK pathway.

**Figure 6:**
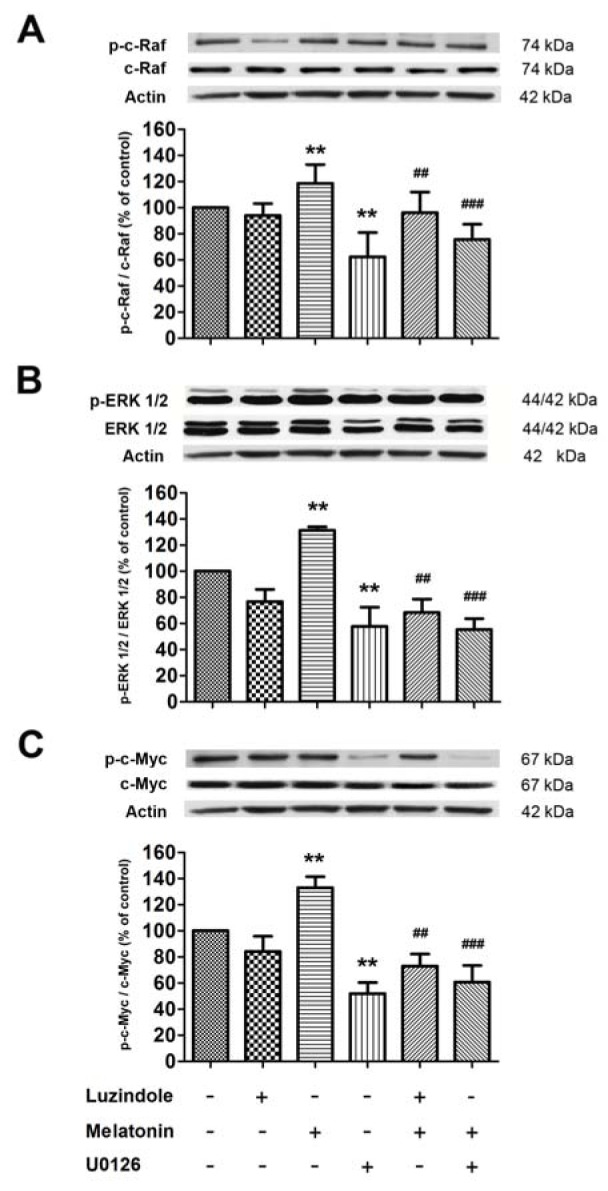
The effect of luzindole, an antagonist of melatonin receptor and U0126, a MEK1/2 selective inhibitor, on the melatonin-induced increase in signaling protein expression. The levels of p-c-Raf, pERK1/2 and p-c-Myc were normalized with c-Raf, ERK1/2 and c-Myc, respectively and quantified using densitometry. ANOVA was performed for statistical analysis and the results are expressed as mean ± S.E.M. of four independent experiments (** denotes statistical significance at P < 0.01 compared with the control; ## and ### denote statistical significance at P < 0.01 and < 0.001, respectively, compared with melatonin-treated cells).

Western blot analysis (Figure 6(Fig. 6)) showed that c-Raf, ERK and c-Myc phosphorylation increased significantly following the administration of 1 µM melatonin, compared with the untreated control group. This activation significantly decreased by pre-incubation with 1 µM luzindole or 1 µM U0126 compared to untreated cell. The present data further indicated that the ERK/MAPK pathway is the possible pathway which regulated SVZ precursor proliferation by melatonin through the melatonin receptor. 

## Discussion

In this examination, we revealed that melatonin could perform as a trophic factor, like EGF or bFGF, to promote adult neural precursor cell proliferation. Although melatonin can enhance neurogenesis in embryos and adults (Kong et al., 2008[27]; Moriya et al., 2007[36]; Sotthibundhu et al., 2010[51]), the precise subsequence, by which melatonin up-regulates cell proliferation, is not fully understood. For further better understanding the pleiotropic function of melatonin, its effects and modes of action are examined at cellular and sub-cellular levels. 

The second messenger cascades that are stimulated by MT1 and MT2 receptors seem to differ in a cell-, tissue- and species-specific manner. The possible cell signaling pathways downstream of the MT receptors include the Rafs/MEK1/2/ERK1/2 pathway, which is stimulated by several mechanisms (Kilic et al., 2005[25]; Luchetti et al., 2009[33]; Radio et al., 2006[40]; Witt-Enderby et al., 2000[58]). In particular, our* in vitro* and *in vivo* studies show that the proliferative outcome of melatonin is occurred via the activation of the ERK/MAPK pathway. As a blotting result, melatonin markedly increases the phosphorylation of c-Raf, ERK1/2 and c-Myc, respectively. The MT1 receptor can also activate PLC in the presence of an enlargement in intracellular calcium, resulting in phosphoinositide hydrolysis and the subsequent stimulation of PKC activity (Baba et al., 2013[4]; Chan and Wong, 2013[9]; von Gall et al., 2002[55]). ERK/MAPK signaling pathways are up-regulated upon the activation of various types of receptors (Balmanno and Cook, 2009[5]; Chan and Wong, 2013[9]; Roudabush et al., 2000[46]; Wells, 1999[57]). We examined the involvement of melatonin receptors in the melatonin-induced activation of the ERK/MAPK signaling pathway whether this signaling pathway underlies the proliferative effect of melatonin on precursor cells from the adult mouse. 

Growth factor signaling is important in stem cell maintenance and function, and typical growth factor pathways utilize receptor tyrosine kinase signaling. EGF can stimulate neural stem cells to self-renew and proliferate in response EGFR (Doetsch et al., 2002[15]; Shi et al., 2008[49]) via the stimulation of the MAPK signaling cascade. In this study, we have shown similar numbers of spheres forming in the EGF and EGF plus bFGF groups. This result was sustained from a previous study which showed that the main growth factor affecting neural stem cell proliferation is EGF (Moriya et al., 2007[36]). Significant increase in proliferation was observed when two growth factors (EGF+bFGF) and melatonin were used simultaneously compared with EGF+bFGF or compared with melatonin alone. This suggested the synergistic effect occurred. In addition, the present result also suggested the synergistic effect of melatonin and growth factors on the activating the ERK/MAPK pathway.

Upon activation, ERK1/2 is translocated to the nucleus and phosphorylate multiplex transcription factors that must be modulated in response to growth factors to control cell cycle progression, proliferation, survival, and the down-regulation of antiproliferative genes (Johnson and Lapadat, 2002[22]; Junttila et al., 2008[23]; Meloche and Pouyssegur, 2007[35]). In addition, activated-ERK1/2 also enhances c-Myc protein stability and plays a principle role in controlling cell cycle progression and apoptosis by driving cells from G0 to late G1 phase (Pelengaris et al., 2002[39]; Sears et al., 2000[47]). Although the cell cycle molecules modulated by melatonin remain to be illustrated, it has been stated that melatonin increases G0/G1 to G2+M cells distribution in epithelial cells (Li et al., 1999[30]) and cyclin A protein expression in breast cancer cells (Margheri et al., 2012[34]). However, melatonin affects the cell cycle presumably occurring by different doses, duration and related to the cell line-specific pattern.

Blocking MEK1/2 with low concentration of antagonist U0126 completely abolished melatonin-induced ERK1/2 phosphorylation in the adult mouse SVZ precursor cells, demonstrating that melatonin acts upstream of ERK1/2 to trigger proliferative signaling. As our results, treatment with low concentration of U0126 alone did not have any effect on the control levels of the proposed activities. ERK/MAPK signaling can be induced by various molecules, one of which is EGF, which is the main growth factor typically used in culture media. In addition, U0126 has been shown not only suppresses MEK1/2 activity, but also indirectly elevates PI3K (Benz et al., 2010[7]; Hayashi et al., 2008[19]). In this respect, ERK/MAPK and PI3K signaling have separated roles at different times in G2/M cell cycle progression (Roberts et al., 2002[43]).

Interestingly, melatonin and MT1 expression were detected in the developing brain (Jimenez-Jorge et al., 2007[21]). This finding suggests that melatonin could perform as a protective mechanism against free radical damage and could exert some trophic activities. In addition, the expression of MT1 receptors during normal brain development indicates that brain melatonin could function along this period. Melatonin has recently been found as a trophic factor in chick brain development, during which it stimulates mitotic activity in diencephalic astrocytes (Paulose et al., 2009[38]). Melatonin treatment can also increase the total of cells with neurite processes in N1E-115 cells, SK-N-SH cells and CHO cells (Benitez-King et al., 1990[6]; Cos et al., 1996[12]; Witt-Enderby et al., 2000[58]), and this effect occurred via the MT1 receptor by activation of phosphorylation of ERK1/2 (Witt-Enderby et al., 2000[58]). 

Our prior study (Sotthibundhu et al., 2010[51]) showed that the precursor cells obtained from the adult mouse SVZ expressed the MT1 receptor and the proliferative effect of melatonin was antagonized by luzindole and prevented by pretreatment with pertussis toxin (PTX). The instant study presented that the stimulatory effect of melatonin on neural stem cell proliferation via melatonin receptor through ERK1/2 activation were further supported by the results showing that luzindole treatment abolished the increase of p-ERK1/2 caused by melatonin. We showed here that melatonin treatment induced the up-regulation of p-c-Raf, p-ERK1/2 and p-c-Myc in differentiated neural stem cells compared with control group both *in vitro* and *in vivo *models.

A pharmacological of melatonin receptor blockage or the ERK1/2 signaling pathway in zebra fish delays neuron differentiation and the formation of neural processes in the habenular nuclei (de Borsetti et al., 2011[14]). This study also showed that the appropriate outgrowth of dendrites during habenular development requires light and melatonin. In addition, some neurological diseases, including autism can be occurred by abnormal melatonin synthesis (Kulman et al., 2000[28]; Leu et al., 2011[29]; Rossignol and Frye, 2011[45]). It has been suggested that melatonin levels during early postnatal development may affect the formation of brain circuits. Crupi et al. (2011[13]) demonstrated a stimulatory role of melatonin in the adult mouse hippocampal neurogenesis while Ramirez-Rodriguez et al. (2011[41]) also expressed that melatonin stimulated dendrite maturation in adult hippocampal neurogenesis of mice.

## Conclusion

In conclusion, this study shows that the stimulatory effects of melatonin on adult mouse SVZ proliferation are mediated through the melatonin receptor, which activates the ERK/MAPK signaling pathway. Stimulated c-Raf, ERK1/2 lead to enhance c-Myc protein (a transcription factor) stability and trigger proliferative signaling. Because of crossing the placental and the blood-brain barriers and soluble in both lipids and water ability (Kilpatrick et al., 1995[26]), this endogenous modulator might be benefit for stimulating local neural stem cell proliferation. The accumulated results suggest that melatonin plays a trophic factor-like regulatory role, which provides new insights into postnatal brain development. In addition, the discovery of the mechanism by which melatonin modulates neural precursor cell proliferation and differentiation may be used to improve innovative strategies for the therapeutics of neurodegenerative diseases.

## Conflict of interest

The authors declare that they have no conflict of interest.

## Acknowledgements

This study was supported by a Mahidol University Postdoctoral Fellowship to AS and research grants from Thailand Research Fund (DPG5780001, IRG5780009) and Mahidol University to PG.
